# Supplementary material for: The PROCAN-B study protocol: Early diagnosis of PROstate CANcer for Black men—a community-centred participatory approach in Scotland and the North-East of England
Source: PLoS One. 2024 Dec 31;19(12):e0315288. doi: 10.1371/journal.pone.0315288 (PMC11687736; doi:10.1371/journal.pone.0315288)
Supplement: Attachment — Submitted filename: Revisions table.docx [file pone.0315288.s001.docx]

| Many thanks to the reviewers for their helpful and constructive comments. Please find here the table outlining the changes made. | |
| --- | --- |
| **Comments** | **Response** |
| 1. Please ensure that your manuscript meets PLOS ONE's style requirements, including those for file naming. The PLOS ONE style templates can be found at  <https://journals.plos.org/plosone/s/file?id=wjVg/PLOSOne_formatting_sample_main_body.pdf> and  <https://journals.plos.org/plosone/s/file?id=ba62/PLOSOne_formatting_sample_title_authors_affiliations.pdf> | Thank you, I believe the manuscript meets the requirements |
| 2. Thank you for stating the following financial disclosure:  "Authors who received this award are: FC, JL, JE, MKo, JK, KR. This study is funded by Prostate Cancer Research <https://www.prostate-cancer-research.org.uk/>  Grant reference number: 6968"  Please state what role the funders took in the study.  If the funders had no role, please state: ""The funders had no role in study design, data collection and analysis, decision to publish, or preparation of the manuscript.""  If this statement is not correct you must amend it as needed.  Please include this amended Role of Funder statement in your cover letter; we will change the online submission form on your behalf. | This statement is correct and this has now been changed in the cover letter. |
|  |  |
| 3. Please include a copy of Table 3 which you refer to in your text on page 17. | Table 3 was already included in the manuscript, this can now be found on page 21 |
| 4. Please review your reference list to ensure that it is complete and correct. If you have cited papers that have been retracted, please include the rationale for doing so in the manuscript text, or remove these references and replace them with relevant current references. Any changes to the reference list should be mentioned in the rebuttal letter that accompanies your revised manuscript. If you need to cite a retracted article, indicate the article’s retracted status in the References list and also include a citation and full reference for the retraction notice.  [Note: HTML markup is below. Please do not edit.]  Reviewers' comments:  Reviewer's Responses to Questions | Thank you, this has been done. Newly added literature has been highlighted in yellow |
| Reviewer #1: Manuscript Feedback  A theoretical framework is provided: Integrated Screening Action Model. However, how this framework informs the study design or other aspects of the study (Focus Group guide, refining intervention testing, etc.) is not mentioned. Sample Size/Recruitment. | Many thanks for this comment. We have tried to make this important element clearer. The I-SAM model is integrated throughout the study. The model is briefly explained at the start of the methods section. A sentence has also been added to this section indicating that the model feeds into data collection, data analysis and ultimately intervention development as the draft intervention strategy will be developed from the I-SAM’s target intervention functions, and policy categories, to improve access to screening. These are taken from the Behaviour Change Wheel (BCW) but we have now made it clearer hopefully that the BCW is integrated in the I-SAM. It is then mentioned in WP2 that the barriers and facilitators found in WP1 will be mapped onto the I-SAM to inform the intervention. WP5 also described how the I-SAM will inform data analysis of the focus groups, and the subsequent intervention refinement in WP6. WP7 is also underpinned by the I-SAM to ensure all relevant concepts, such as capability, opportunity and motivation, are captured in the data collection tools (surveys). A sentence has been added to emphasise this in WP7. References to the BCT taxonomy and the Behaviour Change Wheel have been removed not to cause confusion. |
| Objective 1 – exploration of barriers. Sample group of N=13 for both sites is too small to give diverse views from both communities. | The original proposal underpinning this protocol was peer-reviewed and funded, with a proposed sample size of n=12. Sample sizes in qualitative research are a topic of debate in health research. Qualitative research does not aim to be representative and aims to achieve depth, rather than breadth (Vasileiou et al 2018). The smaller sample size allows for in-depth exploration of perspectives of the target group, and data saturation was the aim rather than representativeness. This is in line with existing qualitative research (Guest et al 2006). As multiple sessions were scheduled with the PICE group, and sessions were run by experienced qualitative researchers, saturation would be a possibility. However, as we did aim to explore wide-ranging perspectives, purposive sampling was used to recruit a diverse PICE group, particularly regarding ethnicity and age. Purposive sampling was applied to ensure inclusion of multiple African ethnicities and as the recruitment leads are both Black African, an effort was made to recruit Black Caribbean men to ensure their voices were included too. Although we aimed to recruit 12 men for the PICE group, we managed to recruit 13. However, as reviewer 2 rightfully pointed out, we did add to the submitted protocol some elements that had been conducted already at the time of submission of the protocol (October 2023), which reviewer 2 did not think was appropriate as part of the protocol. Therefore, we have now stated we aimed for a sample size of 12 and added a justification in line with the above, supported by qualitative literature:  Guest G, Bunce A, Johnson L. How many interviews are enough? An experiment with data saturation and variability. Field Methods. 2006;18(1):59–82.  Vasileiou, K., Barnett, J., Thorpe, S. *et al.* Characterising and justifying sample size sufficiency in interview-based studies: systematic analysis of qualitative health research over a 15-year period. *BMC Med Res Methodol* **18**, 148 (2018). |
| Objective 2 – design of intervention. The sample size of 13 for both sites seem small to capture diverse views, especially given the aim of designing an intervention that is culturally relevant to each site. | As above, the sample size is in line with existing qualitative research. The proposal underpinning this protocol was peer-reviewed and funded on this basis. |
| Objective 6 – Same observation as for Objectives 1 &2 above Objectives 4, 5, 7, 8, the sample size appear more reasonable. | As above, the sample size is in line with existing qualitative research. The proposal underpinning this protocol was peer-reviewed and funded on this basis. |
| Work Package 2. Are the participants involved in this Work Package, the same 13 participants recruited for Work Package 1? If so, how would you ensure some fresh perspectives are brought to the table in terms of potential intervention ideas? How about having a mix of participants who were part of Work Package 1 and some participants who were not? | Yes, these are the same men as they form the study’s PICE (PPI) group. It was appropriate to have the same men in WP1 as in WP2 as WP1 was an exploration of the challenges to early diagnosis of prostate cancer, and WP2 consists of an attempt to tackle those challenges. In work packages (4-8), the intervention is thoroughly evaluated with an additional 60 men potentially offering differing perspectives. It is not pragmatic to include a larger group in the intervention design. Also, the methods in this study protocol cannot be changed now, the proposal was peer-reviewed and funded on this basis, which is what we are aiming to report and disseminate. The PICE group will stay the same throughout the entire study, as is the norm for a PPI/advisory group. |
| Overall Feedback Good proposal with the potential to garner some creative interventions that will be adapted by the targeted communities. | Many thanks for this positive comment, it is much appreciated. |
|  |  |
| Reviewer #2: The "PROCAN-B Study Protocol: Early Diagnosis of Prostate Cancer for Black Men - a community-centered Participatory Approach in Scotland and the North-East of England" aims to address the critical issue of health disparities in prostate cancer diagnosis among Black men through a community-centered, mixed methods research approach. While the protocol robustly outlines the involvement of the community via a Public Involvement and Community Engagement (PICE) group and integrates both qualitative and quantitative research methods, there are several areas where clarity and depth could be enhanced to improve the manuscript's strength and acceptability for publication. These areas include better integration of the mixed methods data, a more streamlined theoretical framework, detailed explanations of sample size justifications, and a more thorough discussion of ethical considerations and the intervention's specifics. Enhancing these aspects will provide clearer insights into the study's methodological rigor and potential impact. Additional comments below: Based on the information provided from the sources, the PROCAN-B study protocol titled "The PROCAN-B Study Protocol: Early diagnosis of PROstate CANcer for Black men - a community-centered participatory approach in Scotland and the North-East of England" is a comprehensive approach designed to address disparities in prostate cancer diagnosis and treatment among Black men. The study employs a mixed methods design and emphasizes community participation and cultural tailoring of interventions, which are its significant strengths. However, for this study protocol to be enhanced and made more acceptable for publication in a peer-reviewed journal, the following areas could benefit from improvements or further clarity: | Many thanks for the constructive comments, we have addressed these below |
| The introduction clearly states the problem and the target population, emphasizing the health disparity for prostate cancer in Black men in the UK. However, it could be enhanced by adding a concise review of the existing literature that highlights gaps the study aims to fill. Providing a brief overview of prior interventions and their limitations can strengthen the rationale for this study. | Yes, agreed. We have done this now and added key literature at the end of the introduction, page 6, line 13-to line 6 page 6 |
| • Multiple theoretical frameworks are introduced (e.g., I-SAM, COM-B, socio-ecological model), but their interrelations and specific contributions to the research questions could be articulated more clearly to avoid conceptual overlap and confusion. I also think the whole protocol is too bloated with unnecessary use of frameworks without adequate justification of use. | Apologies, we agree, this was confusing. As explained above, those frameworks are incorporated into the I-SAM and we have attempted to simplify this now. |
| Additionally, articulating the theoretical framework that guides the study at the outset would lay a solid foundation for understanding the choice of methods and intervention design. | Thank you, agreed. Yes, hopefully we have done this better now |
| • While the socio-ecological conceptual framework is mentioned, its application to the study could be elaborated. How exactly does this framework guide the study's design and the development of interventions? A more detailed explanation would help in linking theoretical underpinnings directly to study actions and expected outcomes. | As above, it’s part of the I-SAM and we have attempted to explain the participant and environmental influences better when explaining the I-SAM (page 7-8) |
| • Several abbreviations were not defined upon first use. | Apologies, we have carefully gone through the manuscript and corrected this where needed. |
| • Expanding on the rationale and benefits of using a participatory and community-centered approach for this specific population could solidify the study's methodological choice. Briefly discussing evidence that supports the effectiveness of such approaches in similar settings would strengthen the argument for adopting this strategy. | We have provided a stronger rational regarding participatory approaches in the introduction (page 5, line 26, page 6 line 7) |
| • There are mentions of the high incidence and mortality rates of prostate cancer among Black men, but we could benefit from more current or specific epidemiological data to underscore the urgency and scale of these disparities. | We have added more recent studies in page 4, line 10-14  Down, L., Barlow, M., Bailey, S.E.R., Mounce, L.T.A., Merriel, S.W.D., Watson, J. and Martins, T., 2024. Association between patient ethnicity and prostate cancer diagnosis following a prostate-specific antigen test: a cohort study of 730,000 men in primary care in the UK. *BMC Medicine,* **22**, pp. 1-10.  6. Delon C, Brown KF, Payne NWS, Kotrotsios Y, Vernon S, Shelton J. Differences in cancer incidence by broad ethnic group in England, 2013–2017. British Journal of Cancer 2022 126:12. 2022;126(12):1765-73.  7. Butler EN, Kelly SP, Coupland VH, Rosenberg PS, Cook MB. Fatal prostate cancer incidence trends in the United States and England by race, stage, and treatment. British journal of cancer. 2020;123(3):487-94. |
| • The introduction asserts the importance of early diagnosis in closing health disparities but does not sufficiently address how early diagnosis can specifically mitigate these disparities. It could be beneficial to include evidence or theories suggesting that earlier diagnosis leads to better treatment outcomes and how these outcomes specifically impact the mortality and morbidity rates among Black men. | We have tried to clarify this in line 22-23 page 4, in the introduction |
| • There is mention of cultural and structural barriers; it would be strengthened by a deeper exploration of these factors. Discussing specific examples or past research findings that illustrate how these barriers manifest and affect health behaviors could provide more context and depth. | We have addressed this on line 24 page 4, to line 9 page 5 by discussing common barriers such as lack of knowledge/awareness, lack of trust, embarrassment and sexuality (fear of homophobia) |
| • The statement about the limited research in the UK on this topic is compelling but could be enhanced by a brief review of what has been done internationally and how this study's approach differs or builds on previous work. | We have addressed this in line 12-17 page 6 explaining some of the evidence from the US |
| • The selection of study locations—Scotland and the North-East of England—raised questions about the demographic relevance, especially since areas like London and the West Midlands have a higher concentration of Black populations in the UK. The protocol mentions these regions are socioeconomically deprived with poor cancer outcomes, which might suggest a strategic focus on under-studied regions. However, for robustness and clarity, this choice warrants a more detailed justification. | The study locations were chosen on purpose and were justified by stating the following: ‘The study will run in two socioeconomically deprived areas with poor cancer outcomes: Scotland and the North-East of England, which lack inclusion in UK research. Including Black men from less ethnically diverse settings, is important to ensure their voices are heard. These two settings also incorporate different healthcare systems, which could be important for future feasibility testing, and are pragmatic choices as they are where the researchers are based’.  Choosing settings in London and the West-Midlands would have been easier, as recruitment would be less challenging, however UK research is biased towards the South of England and including Black men from less ethnically diverse settings, means we will capture voices that are normally not heard. |
| • More specificity about the sampling strategy for focus groups, including how participants are identified and recruited, could enhance transparency and replicability. Detailing the inclusion and exclusion criteria would also fortify this section. • | Inclusion and exclusion criteria are included on page 9  Sampling and recruitment are in detail described on page 9 and 10 |
| The methods section should detail a specific integration strategy, such as a convergent design where both data types are collected simultaneously and analyzed separately, then merged to draw meta-inferences. This should include an explanation of how these inferences are synthesized to create comprehensive insights that neither method could achieve alone. Describing the analytical techniques, such as joint displays or narrative weaving, will further illustrate how data interrelate and support the study's conclusions. | A description of the mixed-methods exploratory sequential design (based on Creswell) has been added to the research design section on page 8, line 4-8, as well as a short description of the integration of data on page on 24, line 6-7 |
| While the method for thematic analysis using NVivo is mentioned, the protocol could benefit from a more detailed description of the coding process, including how codes will be derived and how discrepancies between coders will be handled. | The different steps in coding are described on page 21 and Braun & Clarke’s thematic analysis is followed. Line 14-15 on page 22 and 3-4 on page 23 have been added to offer more insight into the coding and how disagreements will be solved. |
| • The intervention's development is participatory, which is a strength. Yet, the description of how feedback from the community is systematically incorporated into the intervention development could be more explicit. • | More detail on this process has been added in WP2, page 14, line 17-25 |
| The use of the Integrated Screening Action Model (I-SAM) is well-noted, but the manuscript could benefit from a more detailed explanation of how this model interacts with other theoretical frameworks mentioned, such as the COM-B model and the socio-ecological model. Clarifying how these models collectively inform the research questions, design, and expected outcomes could strengthen the theoretical basis. | Please see above |
| o Also, while the I-SAM is intended to guide intervention development, specific examples of how its components (capability, opportunity, motivation) are operationalized within the study’s interventions would help in translating theory into practice more clearly. | This has now been added in the description of the I-SAM on page 7 and 8. We’ve also decided to add the I-SAM model as Figure 1, which will help to present the model. |
| • Each work package is aligned with specific objectives, but expanding on how these packages integrate with each other to achieve the overall study goals could clarify the step-by-step progression of the research. | Thank you, the progressive nature has now been more explicitly explained on page 12, line 19-23 |
| o Also some sample sizes in the table do not match the descriptive texts. | Thank you, this has now been checked. |
| • No detailed rationale for the chosen sample sizes in each work package. It's essential to clarify why specific numbers were selected and how they ensure statistical power or adequacy for meaningful analysis, especially considering the diversity and complexity of the population being studied. • | Sample size has been justified on page 10, line 11-16 |
| The protocol should specify whether the same participants are used across different WPs or if new participants are recruited for each phase. | Thank you. These are different men (apart from the PICE group), which is more clearly explained now on page 10, line 11-12 |
| o A clear flow diagram or a detailed description of participant progression through the study’s phases would aid in understanding how individuals move through the intervention, evaluation, and refinement stages | A flow diagram showing the study’s phases is included (figure 2) |
| • More detail on the specific methods used in the evaluation phases (WP5 and WP8) and how findings from each phase feed into the next would underscore the iterative nature of the intervention development process. | Some more detail has been added, particularly in WP8. WP5 describes how the initial delivery of the intervention (WP4) will be evaluated, the theoretical framework used to underpin the evaluation and the method of evaluation. WP6 then describes how in collaboration with the PICE group, findings from WP5 will be used to refine the intervention. WP7 describes how the refined intervention will be pilot tested. WP8 describes how the evaluation takes a similar approach as in WP5, which will aid future research and implementation. |
| Pg 8: what does different ethnicities mean? | We have specified on page 10 line 4-5: including a diverse sample of men born in the UK and/or of African origin from various countries across the African continent, as well as from the Caribbean. |
| • How would findings from these areas be extrapolated or related to Black men in other UK regions? | Findings will not be generalisable however it is discussed in the discussion on page 25 line 12-21 that detailed descriptions of methods will be provided which could make findings transferable. |
| What exactly are the community engagement efforts that informed the choice of these locations. If local organizations or healthcare providers have existing relationships or have identified specific needs, these details would strengthen the rationale for site selection. | It is explained on page 11 that two members of the community, who are also part of community organisations, are employed as Recruitment leads on the project. We have added that these community organisations have agreed to collaborate with the study. |
| • Since this is a protocol, expected results should not be discussed, so it’s confusing why there are some results presented from some of the work packages. | Thank you for this useful comment, agreed. This has now been corrected in WP1. |
| DISCUSSION • The protocol could pre-emptively address how it intends to interpret and disseminate the findings, especially in light of potential limitations. How does the study's design (including its participatory elements) impact the generalizability of the results to other settings or populations? • What are the expected contributions to the literature, potential implications for practice and policy, and future research directions? | Thank you, dissemination and generalisability have now been addressed in the discussion on page 25, line 6-21  The expected contribution has been addressed on page 24, starting line 28 running through to the dissemination and discussion of transferability. |
